# The distinct biological implications of *Asxl1* mutation and its roles in leukemogenesis revealed by a knock-in mouse model

**DOI:** 10.1186/s13045-017-0508-x

**Published:** 2017-07-11

**Authors:** Yueh-Chwen Hsu, Yu-Chiao Chiu, Chien-Chin Lin, Yuan-Yeh Kuo, Hsin-An Hou, Yi-Shiuan Tzeng, Chein-Jun Kao, Po-Han Chuang, Mei-Hsuan Tseng, Tzu-Hung Hsiao, Wen-Chien Chou, Hwei-Fang Tien

**Affiliations:** 1 Graduate Institute of Clinical Medicine, National Taiwan University, Taipei, Taiwan; 2 Department of Medical Research, Taichung Veterans General Hospital, Taichung, Taiwan; 3 Department of Laboratory Medicine, National Taiwan University Hospital, No. 7, Chung-Shan S Rd, Taipei, 10002 Taiwan; 4 Graduate Institute of Oncology, College of Medicine, National Taiwan University, Taipei, Taiwan; 5 Division of Hematology, Department of Internal Medicine, National Taiwan University Hospital, No. 7, Chung-Shan S Rd, Taipei, 10002 Taiwan

**Keywords:** Asxl1, MN1, Hematopoietic stem cell, Engraftment

## Abstract

**Background:**

*Additional sex combs-like 1* (*ASXL1*) is frequently mutated in myeloid malignancies. Recent studies showed that hematopoietic-specific deletion of *Asxl1* or overexpression of mutant *ASXL1* resulted in myelodysplasia-like disease in mice. However, actual effects of a “physiological” dose of mutant *ASXL1* remain unexplored.

**Methods:**

We established a knock-in mouse model bearing the most frequent *Asxl1* mutation and studied its pathophysiological effects on mouse hematopoietic system.

**Results:**

Heterozygotes (*Asxl1* ^*tm/+*^) marrow cells had higher in vitro proliferation capacities as shown by more colonies in cobblestone-area forming assays and by serial re-plating assays. On the other hand, donor hematopoietic cells from *Asxl1* ^*tm/+*^ mice declined faster in recipients during transplantation assays, suggesting compromised long-term in vivo repopulation abilities. There were no obvious blood diseases in mutant mice throughout their life-span, indicating *Asxl1* mutation alone was not sufficient for leukemogenesis. However, this mutation facilitated engraftment of bone marrow cell overexpressing *MN1*. Analyses of global gene expression profiles of *ASXL1*-mutated versus wild-type human leukemia cells as well as heterozygote versus wild-type mouse marrow precursor cells, with or without *MN1* overexpression, highlighted the association of in vivo *Asxl1* mutation to the expression of hypoxia, multipotent progenitors, hematopoietic stem cells, *KRAS*, and *MEK* gene sets. ChIP-Seq analysis revealed global patterns of *Asxl1* mutation-modulated H3K27 tri-methylation in hematopoietic precursors.

**Conclusions:**

We proposed the first *Asxl1* mutation knock-in mouse model and showed mutated *Asxl1* lowered the threshold of *MN1*-driven engraftment and exhibited distinct biological functions on physiological and malignant hematopoiesis, although it was insufficient to lead to blood malignancies.

**Electronic supplementary material:**

The online version of this article (doi:10.1186/s13045-017-0508-x) contains supplementary material, which is available to authorized users.

## Background

*Additional sex combs-like 1* (*ASXL1*) is the human homolog of Drosophila *additional sex combs* (*Asx*) [1], frequently mutated in acute myeloid leukemia (AML) and other myeloid malignancies [2–4]. Germline heterozygous nonsense mutation of *ASXL1* results in Bohring-Opitz syndrome, a congenital disease with multi-system developmental abnormalities [5]. ASXL1 binds a deubiquitinase BAP1 to form a critical complex for H2A K119 deubiquitination through the catalysis of polycomb repressive complex 1 [6, 7]. The deubiquitination activity is enhanced when BAP1 is complexed with truncated form of ASXL1 [8]. BAP1 deletion produces phenotypes mimicking human chronic myelomonocytic leukemia in mice [9]. Thus, it is likely that ASXL1-BAP1 axis is important to prevent leukemogenesis [9].

We previously analyzed the clinical implications of *ASXL1* mutation in a large cohort of patients and found that this mutation occurred in 10.8% (54/501) of de novo AML patients and predicted a shorter survival [10]. Several studies also showed that *ASXL1* mutation was a poor prognostic factor in myeloid malignancies [10–14].

Since the discovery of *ASXL1* mutation in myeloid malignancies in 2009 [15], many studies about its pathophysiology have been reported. However, controversies exist among these reports. For example, in vivo deletion of *Asxl1* was shown to result in subtle phenotypes including defects in the frequencies of myeloid and lymphoid cells in blood, marrow or other hematopoietic organs in mice but not myelodysplastic syndrome (MDS) or leukemia [16]. However, in other studies, knockout of *Asxl1* led to systemic developmental defects including MDS-like presentation, with alteration of the self-renewal and repopulation capacities of the mutant hematopoietic stem/progenitor cells and global reduction of H3K27 tri-methylation (H3K27me3) [17, 18].

The pathophysiological effect of *ASXL1* truncation mutations in human myeloid malignancies is another matter of debate. For example, it was suggested that *ASXL1* mutation was a loss-of-function mutation because of failure in detecting mutant protein in human leukemia cells [14]. However, the findings that overexpression of truncating mutation in hematopoietic cells of mice displayed human MDS features with de-repression of *Hoxa9* in another study [19] and detectability of truncating proteins in human cell lines bearing *ASXL1* truncating mutations argued for gain-of-function or dominant negative effects of *ASXL1* mutations [19, 20]. These controversies are likely due to different methods of genetic engineering of the animals or forced overexpression of the mutation. Overall, the pathophysiological alterations in human acute myeloid leukemia (AML) cells bearing *ASXL1* mutations have not been explored systematically.

To overcome these problems, we generated and analyzed a mouse model bearing human-like *Asxl1* mutation followed by extensive phenotypic and molecular characterizations on this mouse model. In our model, the *Asxl1* mutation was knocked in to the endogenous *Asxl1* allele, thus the mice have “physiological dose” of mutation, as we see in the patients. For translating to clinical situations, we also investigated the global expression profiles of our large AML cohort to delineate the pathophysiology related to *ASXL1* mutations. We found that bone marrow cells from *Asxl1* heterozygotes formed more colonies in cobblestone-area-forming assays and the ability to form colonies persisted longer in serial colony-forming cell assays. On the other hand, in vivo transplantation assays showed that donor bone marrow cells from *Asxl1* mutant mice declined faster in their recipients than those from the wild-type mice. While forced overexpression of mutant *Asxl1* in mouse bone marrow hematopoietic cells could lead to MDS-like disease [19], our mice bearing a “physiological dose” of mutant *Asxl1* did not show obvious trend of developing blood diseases throughout their life span. However, with overexpression of *MN1*, mutant *Asxl1* hematopoietic stem cells and progenitors (HSPCs) were more likely to engraft in recipient mice than wild-type HSPCs, suggesting that *Asxl1* mutation could lower the threshold of engraftment driven by *MN1* overexpression. Global expression profiling in mutant versus wild-type *Asxl1* mouse cells as well as in *ASXL1*-mutated versus wild-type human AML cells, with or without concurrent *MN1* overexpression, disclosed pathophysiological pathways involved in *Asxl1* mutation. ChIP-Seq experiments showed global *Asxl1* mutation-modulated H3K27me3 patterns in HSPCs.

## Methods

### Generation of *Asxl1* mutation knock-in mice

The cognate mouse mutation is predicted to be c.1925dupG; p.G643WfsX12, encoding 654 amino acids mimicking the most common form of human mutant ASXL1 protein, compared to 1514 residues in wild-type Asxl1 protein. Potential chimeras were crossed with wild-type C57BL/6 mice to facilitate the confirmation of germ-line transmission, their offspring who harbored *Asxl1* mutation were backcrossed with C57BL/6 to generate inbred strains then maintained at C57BL/6 background. Heterozygous mice were mated with wild-type mice to get heterozygous mice and littermate control mice. Heterozygous mice were mated with each other to get homozygous mice. Mice between 2- to 6-month age were used for experiment except those were assigned to long-term observation cohort. All animals were housed in specific pathogen-free animal facility and all procedures were approved by IACUC of the National Taiwan University College of Medicine.

### Chromatin immunoprecipitation sequencing (ChIP-Seq)

We used Lin^-^ bone marrow cells as a surrogate to identify genome-wide histone modification affected by *Asxl1* mutations. Chromatin lysate was harvested and sonicated with a sonicator (Bioruptor®Pico) to shear the DNA into a length ~200 bp, then it was hybridized with anti-H3K27me3 (Millipore, Germany). Immunoprecipitated DNA was sent to the National Center for Genome Medicine and sequenced by Illumina HiSeq 2000 sequencer with 100 × 2 bp paired-end sequencing.

### ChIP-Seq data analysis

Sequencing reads were aligned to the mm10 mouse reference genome by Burrows-Wheeler Alignment tool (BWA; version 0.7.15). We used the Model-based Analysis of ChIP-Seq tool (MACS2) to detect peaks of reads between sample and input sequences in *ASXL1* ^*tm/+*^ and wild-type bone marrow cells. *ASXL1* ^*tm/+*^ (or wild-type) specific peaks were called by intersecting the identified peaks with BEDTools v2.17.0 [21]. These condition-specific peaks were annotated by Peak Annotation and Visualization (PAVIS) and analyzed for the enrichments in gene regions [22]. To realize the biological functions governed by the interaction of *Asxl1* mutation and H3K27me3, we analyzed peaks-associated genes by The Database for Annotation, Visualization and Integrated Discovery (DAVID) v6.8 with default settings [23, 24]. Furthermore, we performed motif analysis on sequences around the condition-specific peaks (±250 bps from peak center) by MEME-ChIP web tool included in the MEME Suite [25, 26]. Sequencing reads and identified peaks were visualized with Integrative Genome Viewer (IGV) [27, 28].

### Statistical analysis

In vitro and in vivo experiments were performed at least three times independently. Data were processed in Microsoft Excel or GraphPad Prism software. Student’s *t* test, paired *t* test, ANOVA or chi-square test were used to compare the differences in values between groups.

For the other experimental procedures, please see the Additional file 1.

## Results

### Generation of *Asxl1* G643WfsX12 gene knock-in mice

**Fig. 1:**
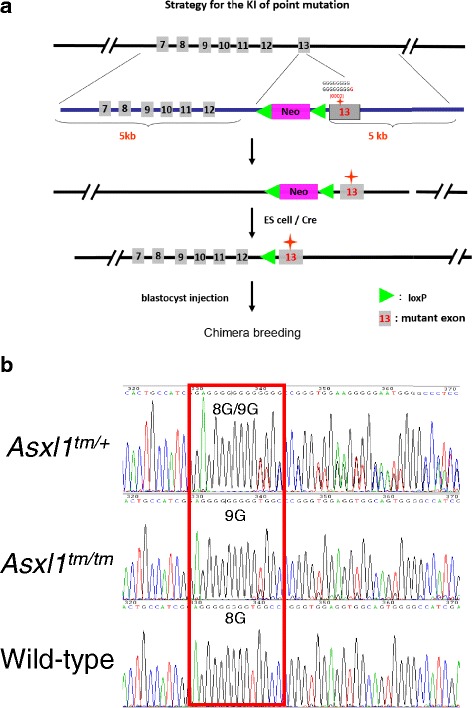
Generation of the *Asxl1* G643WfsX12 knock-in mice. **a** Schematic illustration of the generation strategies of the knock-in mouse model. **b** DNA sequences of *Asxl1* G643WfsX12 heterozygous, homozygous and wild-type bone marrow cells. *Asxl1* mutant had an additional guanine inserting into the 8-G cassette leading to *Asxl1* G643WfsX12 mutation. Since cells with heterozygous *Asxl1* mutant had both 8-G wild-type and 9-G mutant alleles, there were overlapping signals caused by 9-G mediated frame shift after the 8-G/9-G cassette

In human AML, the most common mutation is c.1934dupG; p.G646WfsX12 (up to 66%) [10]. The mouse and human ASXL1 proteins share 74% identity in amino acid sequence. The changed amino acid G646 is within a stretch of highly conserved region ATTAIGGGG**G**PGGGG (designated as a bold and underlined G) [10]. An additional guanine was inserted into this 8-G cassette located at mouse *Asxl1* exon 13 to mimic this frequent human *ASXL1* mutation (Fig. 1a). This insertion causes frame shift in the reading frame and introduces a premature stop codon so that the mutant *Asxl1* would be shorter than wild-type form and lack the c-terminal region which contains a plant homeodomain (PHD). Therefore, the cognate mouse mutation c.1925dupG; p.G643WfsX12 is expected to bear similar pathophysiological consequence as human’s. In our mouse model, mutant *Asxl1* expression was driven by the endogenous *Asxl1* promoter. Therefore, mutant *Asxl1* would be expressed identically as the endogenous *Asxl1*, not restrictive to hematopoietic cells (Fig. 1a).

The additional guanine inserted into the 8-G cassette at exon 13 was confirmed by DNA sequencing (Fig. 1b). Both *Asxl1* G643WfsX12 heterozygotes and homozygotes were fertile. The pups’ genotypes fit Mendelian ratio through gestation period till birth (Additional file 1: Figure S1). Homozygous new-born mice suffered from high rate of post-natal death, with only 7% of viability after weaning. Autopsy of the dead new born mice did not show any obvious organ abnormalities (data not shown). Lack of nursing was probably the main reason of these post-natal lethal events, but the exact causes remains to be elucidated. Due to the difficulty in gathering sufficient mice for observation, we hence focused our long-term observation on heterozygous and wild-type mice.

### *Asxl1*^*tm/+*^ hematopoietic cells had higher short-term in vitro proliferation capacities

**Fig. 2:**
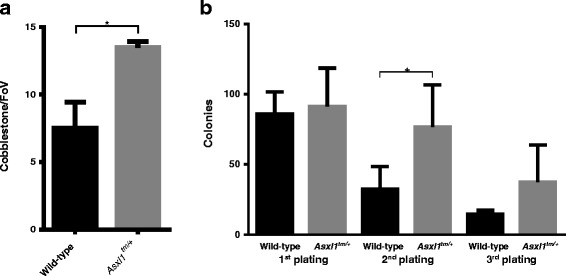
In vitro assays to compare the numbers and potencies of HSPCs between the *Asxl1* G643WfsX12 heterozygous mice and wild type controls. **a** Cobblestone-area-forming cell assays on bone marrow cells from *Asxl1* ^*tm/+*^ mice showed more colonies than those from wild-type control mice (*N* = 3 each). **b** Colony-forming cell assays and serial plating assays showed no significant difference in the ability of forming colonies and differentiation into different lineages in the first plating between *Asxl1* ^*tm/+*^ bone marrow cells and wild controls. However, in the second plating *Asxl1* ^*tm/+*^ cells formed more colonies than wild-type controls. This trend lasted to the third plating but only two wild-type samples had sufficient cells for the third plating, so we were unable to perform t test in the third plating (first plating: wild-type *N* = 5, *Asxl1* ^*tm/+*^ *N* = 7; second plating: wild-type *N* = 5, *Asxl1* ^*tm/+*^ *N* = 6; third plating: wild-type *N* = 2, *Asxl1* ^*tm/+*^ *N* = 6). *p < 0.05

We initially performed several in vitro assays to evaluate the population frequency and differentiation potencies of HSPCs in the bone marrow of *Asxl1* ^*tm/+*^ and wild-type mice. We first used cobblestone-area forming cell (CAFC) assays to evaluate the frequencies of hematopoietic precursor cells in bone marrows. The cobblestone areas were counted one week after seeding and we found that *Asxl1* ^*tm/+*^ cells formed more cobblestone areas than *Asxl1*-wild cells (*N* = 3 each, *p* = 0.028) (Fig. 2a). Colony-forming cell (CFC) assay were also performed to test the effects of *Asxl1* mutant on cell differentiation. Serial plating was performed every 7 days to estimate population frequencies of hematopoietic precursors in the initial plating. In initial plating, *Asxl1* ^*tm/+*^ and wild-type bone marrow cells formed similar numbers of each type of colonies. However, total colony number as well as granulocyte colonies (CFU-G) formed by *Asxl1* ^*tm/+*^ cells were more than wild-type cells in second plating and this trend last to the third plating (Fig. 2b, Additional file 1: Figure S2A to S2C). These results indicate that mutated *Asxl1* confers stronger short-term in vitro proliferation capabilities to hematopoietic precursors than the wild-type *Asxl1*.

### Bone marrow cells of *Asxl1* G643WfsX12 heterozygotes showed compromised long-term in vivo repopulation and self-renewal capabilities

**Fig. 3:**
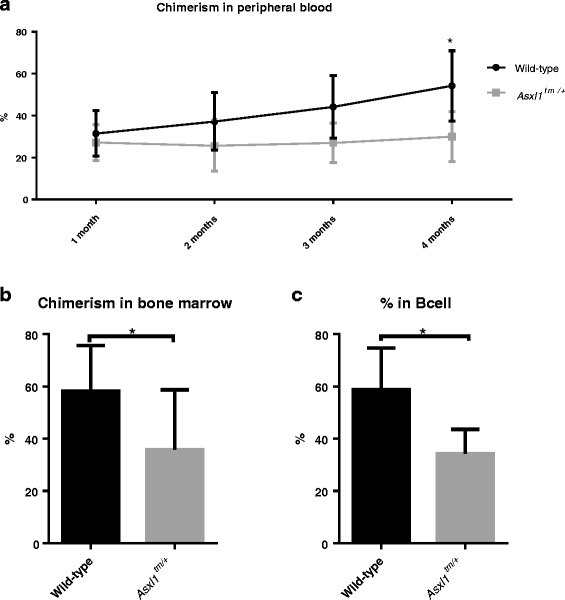
Competitive repopulation assays. The peripheral blood **a** and bone marrow **b** of mice receiving *Asxl1* ^*tm/+*^ cells showed lower chimerism than those receiving wild-type cells in a 4-month observation period. **c** At 4 months after transplantation, donor-derived B cells in the peripheral blood of recipient mice receiving *Asxl1* ^*tm/+*^ cells were particularly reduced compared with those receiving wild-type donor cells

To evaluate the in vivo influence of *Asxl1* mutation on hematopoiesis in a long-term basis, we employed bone marrow transplantation assays. Five hundred Lin^-^c-Kit^+^Sca-1^+^ (LSK) bone marrow cells sorted from *Asxl1* mutant and wild-type mice, respectively, together with 200,000 helper cells, were transplanted into wild-type recipient mice for competitive repopulation unit assays. The peripheral blood of the transplanted mice was sampled monthly and evaluated for reconstitution efficiency in a 4-month period. We found that recipient mice transplanted with LSK bone marrow cells from *Asxl1* ^*tm/+*^ donors had less donor-derived cells in peripheral blood and marrow when compared to those receiving wild-type LSK bone marrow cells (Fig. 3a). Interestingly, B cells in the peripheral blood of *Asxl1* ^*tm/+*^ mice were particularly reduced (Fig. 3c). These data suggest that *Asxl1* mutant LSK cells have reduced in vivo long-term repopulation capacities compared with wild-type LSK cells.

**Fig. 4:**
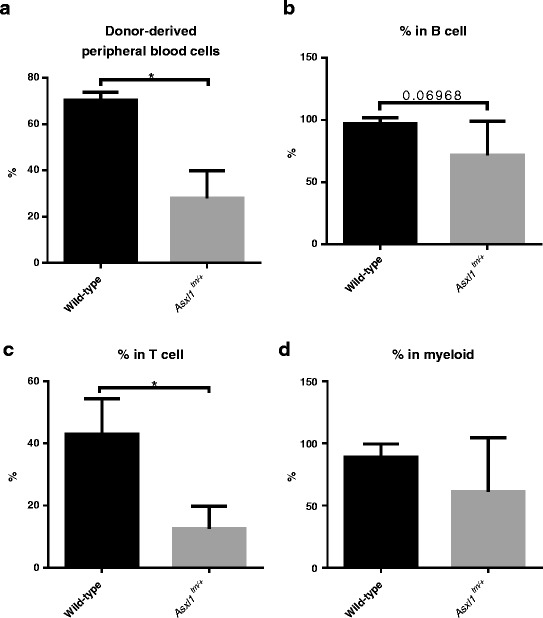
Serial bone marrow transplantation assays. At 2 months after secondary transplantation, the chimerism of total blood cells (**a**) B cells (**b**) T cells (**c**) and myeloid cells (**d**) in the peripheral blood of recipients receiving *Asxl1* ^*tm/+*^ cells declined faster than wild-type donors. *p < 0.05

Next, we performed serial bone marrow transplantation assays to rigorously test the potency of in vivo self-renewal ability of the *Asxl1* ^*tm/+*^ HSPCs. In this setting, whole bone marrow cells were serially transplanted into recipients without helper cells. To evaluate the reconstitution efficiency, peripheral blood of the recipient mice transplanted with either *Asxl1* ^*tm/+*^ bone marrow cells or wild-type bone marrow cells were sampled 2 months after every round of transplantation. We found that the frequencies of total cells and T cells, but not B or myeloid cells, in the recipient mice’s peripheral blood derived from *Asxl1* ^*tm/+*^ mice declined faster compared with those derived from wild-type controls (Fig. 4). The results suggest that *Asxl1* mutation renders a compromised long-term in vivo self-renewal capability in a variety of lineages compared to wild-type cells in vivo.

### The HSPC components of *Asxl1* G643WfsX12 heterozygous mice were largely similar to those of the wild-type littermates

The amount of HSPCs in the bone marrow of *Asxl1* ^*tm/+*^ and wild-type littermates were analyzed and compared by FACS analysis. We noted that bone marrow LSK cells, long-term (Lin^-^Sca-1^+^c-Kit^+^CD150^+^CD48^-^) and short-term hematopoietic stem cells (Lin^-^Sca-1^+^c-Kit^+^CD150^+^CD48^+^), multipotent progenitors (Lin^-^Sca-1^+^c-Kit^+^CD150^-^CD48^+^), common myeloid progenitors (CMP, Lin^-^Sca-1^-^c-Kit^+^CD34^+^FcγR^lo^), granulocyte-monocytic progenitors (GMP, Lin^-^Sca-1^-^c-Kit^+^CD34^+^FcγR^hi^), and megakaryocyte-erythroid progenitors (MEP, Lin^-^Sca-1^-^c-Kit^+^CD34^-^FcγR^lo^) were all not different between the *Asxl1* ^*tm/+*^ heterozygotes and the wild-type mice (Additional file 1: Figure S2D). These data suggest that *Asxl1* mutation does not affect the amount of hematopoietic cell components *in vivo* by surface marker analysis, although both in vitro and in vivo experiments indicate presence of its biological activities in *Asxl1* ^*tm/+*^ bone marrow cells as shown above.

### *Asxl1* G643WfsX12 alone was not sufficient for development of blood malignancies in mice

**Fig. 5:**
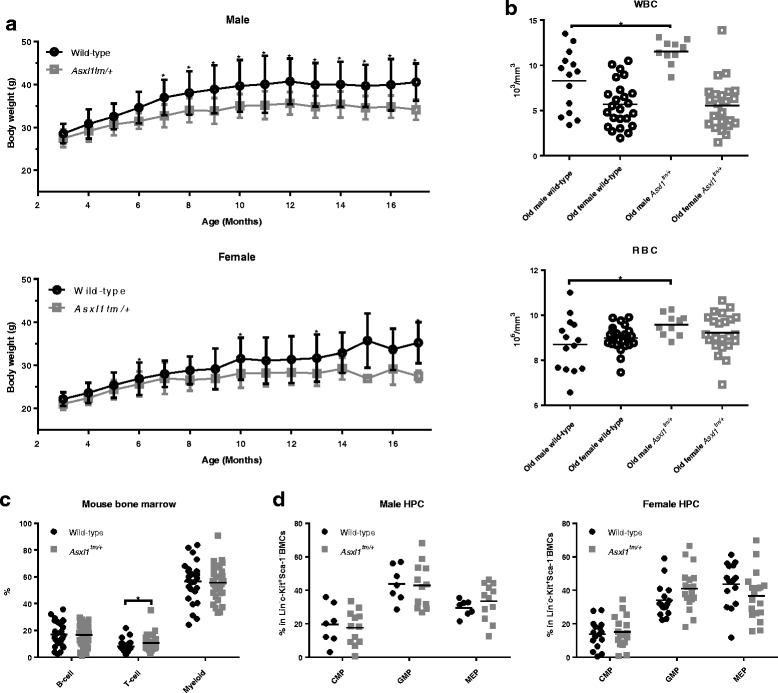
The body weights and hemograms of old *Asxl1* mutant mice (18 months old). **a** Body weights of *Asxl1* heterozygous mice were significantly lower than wild-type mice. **b** Old male *Asxl1* ^*tm/+*^ mice tended to have higher RBC and WBC counts in peripheral blood. **c** There were higher percentages of T cells in old *Asxl1* ^*tm/+*^ mice while both mice had similar frequencies of B cells and myeloid cells in the bone marrow. **d** There was no significant difference in CMP, GMP, and MEP between *Asxl1* ^*tm/+*^ and wild-type control mice. *p < 0.05

A cohort of *Asxl1* ^*tm/+*^ and wild-type control mice were collected to observe the influence of *Asxl1* mutation on overall health status in an 18- to 24-month period (*N* = 33 for *Asxl1* ^*tm/+*^ mice and *N* = 38 for wild-type controls). Heterozygous mice were significantly lighter than wild-type mice (Fig. 5a). While there were no significant differences in hemograms in the peripheral blood or marrow hematopoietic components in younger mice, there were subtle hematopoietic phenotypes when the mice were old at 18 months. Old male (18-month age) *Asxl1* ^*tm/+*^ mice had higher WBC (*p* = 0.0091) and RBC counts (*p* = 0.03893) (Fig. 5b). There were more T cells in bone marrows of *Asxl1* ^*tm/+*^ mice (*p* = 0.049) than wild-type controls, while both mice had similar frequency of HSPCs (Fig. 5c, Additional file 1: Figure S3). Within the Lin^-^c-Kit^+^Sca-1^-^ (LK) bone marrow cells, there was no significant difference in the proportion of CMP, GMP, and MEP between *Asxl* ^*tm/+*^ and wild-type mice (Fig. 5d). The autopsy also did not show any difference in the incidence of splenomegaly between the two groups. The only six viable *Asxl1* homozygous mice did not exhibit obvious abnormalities in hemogram or in autopsy findings at age of 18 months (Additional file 1: Figure S4). During the life span of the mice, *Asxl1* G643WfsX12 showed no tendency to induce any kind of blood malignancies; hence, we concluded that *Asxl1* G643WfsX12 alone was not sufficient for leukemogenesis in vivo.

### *Asxl1* G643WfsX12 lowered the engraftment threshold of *MN1*-overexpressing cells

**Fig. 6:**
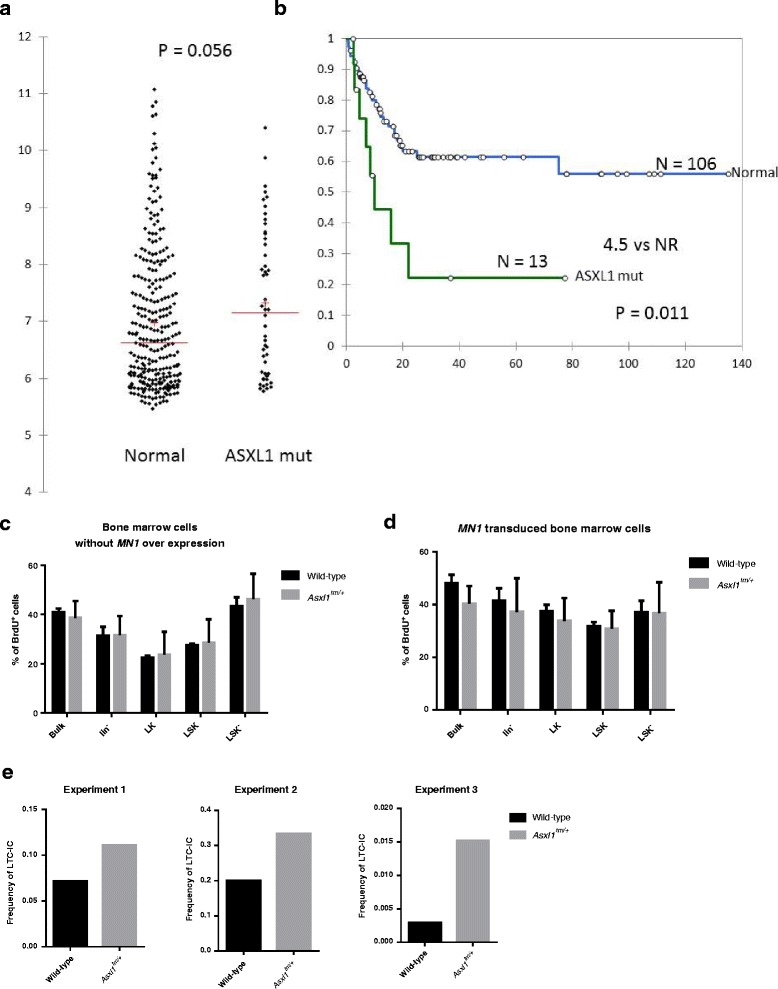
Interaction between mutant *Asxl1* and *MN1* overexpression. **a** According to our patient data, *ASXL1* mutation was associated with higher *MN1* expression. **b** Among patients with higher *MN1* expression, those with *ASXL1* mutation had shorter overall survival than those without this mutation (median 4.5 months vs. not reached). **c** *Asxl1* ^*tm/+*^ had no impact on proliferation of bone marrow cells. **d** *Asxl1* ^*tm/+*^ and wild-type bone marrow cells overexpressed *MN1* did not show significant difference in proliferation. **e** Three independent LTC-IC assays showed that under *MN1* overexpression background, *Asxl1* ^*tm/+*^ bone marrow cells had higher frequency of long-term culture initiation cells than wild-type controls

Since *Asxl1* mutation alone did not produce obvious blood diseases in mice, we sought to determine if this mutation functions as a facilitator for leukemogenesis. In our patients with array data (*N* = 349, among whom the mutation status of *ASXL1* was known in 343) [29–31], we noted that those bearing *ASXL1* mutation tended to have higher *MN1* expression (*P* = 0.056, Fig. 6a). Moreover, among the 225 patients who received standard chemotherapy, those with higher *MN1* expression (≥ median) as well as *ASXL1* mutation had shorter overall survival compared to those with higher *MN1* expression but without *ASXL1* mutation (Fig. 6b), suggesting a possible cooperative effect between these two genetic aberrancies in AML patients. *MN1* over-expression is a sufficient driving event for mouse leukemogenesis [32]. Therefore, we were interested to know whether *Asxl1* mutation facilitates the engraftment of *MN1* over-expression in mice. To this end, we overexpressed *MN1* in *Asxl1* ^*tm/+*^ or wild-type Lin^-^ bone marrow cells by retroviral transduction. Proliferation of wild-type and *Asxl1* ^*tm/+*^ cells were not different as examined by BrdU incorporation assays (Fig. 6c). When *MN1* was transduced into *Asxl1* ^*tm/+*^ and wild-type bone marrow cells, respectively, we still could not observe significant difference in proliferation rate between these two types of cells (Fig. 6d). However, long-term culture-initiation cell (LTC-IC) assay showed that when *MN1* was overexpressed in *Asxl1* ^*tm/+*^ bone marrow cells, there was higher percentage of long-term colony forming cells compared to *MN1* overexpressed wild-type bone marrow cells (Fig. 6e), implying that *Asxl1* mutation promoted stem cell activities of marrow cells in *MN1* overexpression background. To test this hypothesis, we transplanted several different doses of *MN1-*transduced Lin^-^ bone marrow cells, together with 200,000 helper cells, into lethally irradiated recipients. Bone marrow cells of the recipient mice were harvested between 4 to 5 weeks after transplantation to evaluate the reconstitution efficiency. More than 1% *MN1* over-expressing cells in the marrow cells of recipient mice was defined to be successfully reconstituted. At 5000-cell dose, 100% of recipient mice transplanted with either *MN1* overexpressed *Asxl1* mutant or wild-type bone marrow cells were successfully reconstituted. However, while most recipient mice transplanted with low-dose *MN1*-transduced cells could be reconstituted in the presence of *Asxl1* mutation (9 out of 11 at 1000 test cells and 7 out of 7 at 500 test cells were successfully reconstituted), significantly lower proportion of recipient mice transplanted with the same doses of *MN1*-transduced cells without *Asxl1* mutation were successfully reconstituted (7 out of 12 at 1000 test cells and 1 out of 6 at 500 test cells, *p* = 0.036 by Chi-square test) (Table 1 and Additional file 1: Figure S5). Our results suggest that *Asxl1* G643WfsX12 can lower the threshold of *MN1*-driven engraftment.

**Table 1:** The number of mice successfully reconstituted in transplantation assay of *MN1* overexpressed cells with WT or mutant *Asxl1*

Cell dose	WT + *MN1*		*Asxl1* ^*tm/+*^+*MN1*	
	Number tested	Number reconstituted	Number tested	Number reconstituted
5000	6	6	8	8
1000	12	7	11	9
500	6	1	7	7

*p* = 0.036 by chi square test

*Abbreviations*: *WT Asxl1* wild type, *MN1* MN1 overexpression

### Microarray analyses showed the cooperative effects of *Asxl1* mutation and *MN1* overexpression

**Fig. 7:**
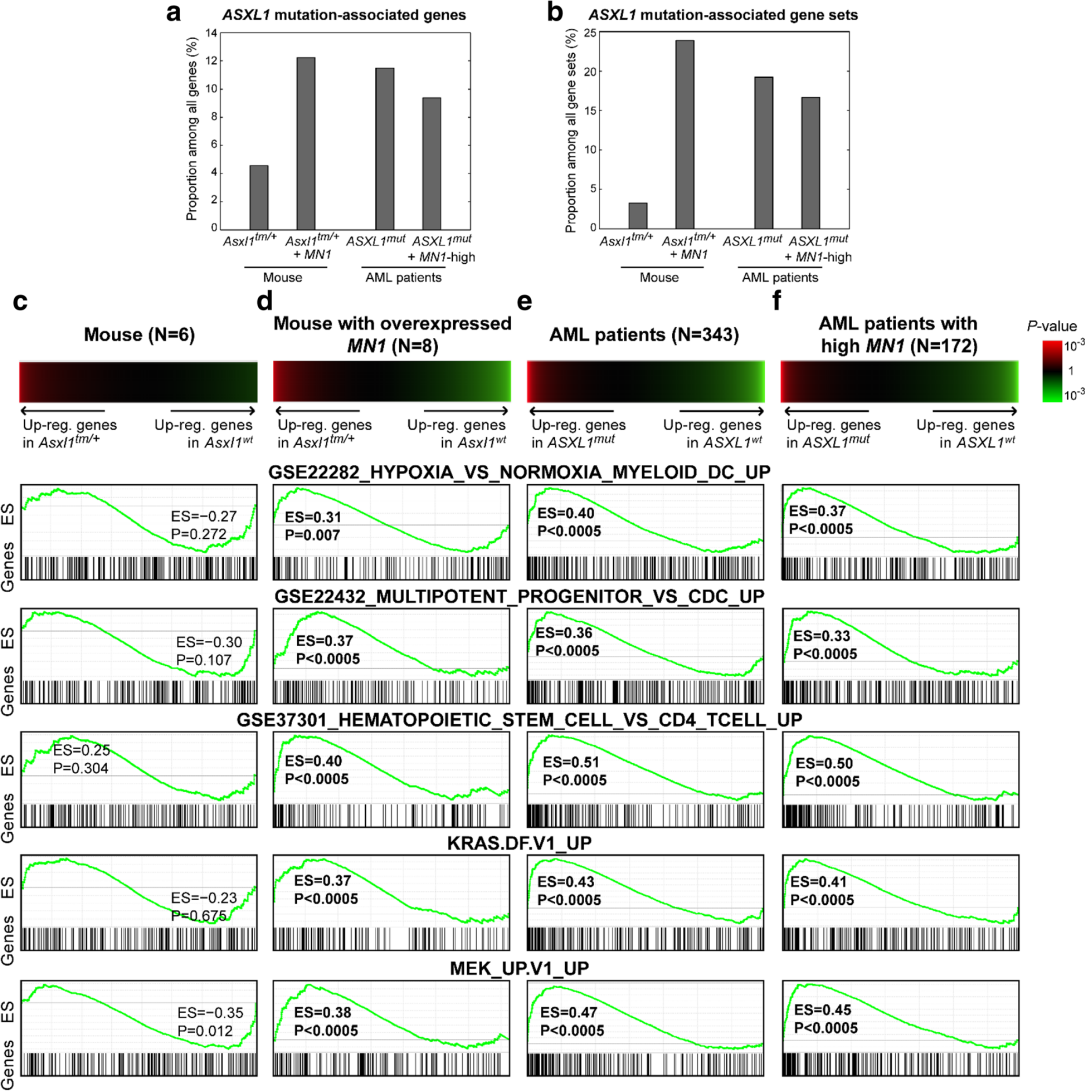
Gene expression and gene set analyses of the interplay between *ASXL1* mutations and *MN1* overexpression. **a** Proportions of significantly differentially expressed genes and **b** enriched gene sets between samples with mutant and wild-type *ASXL1* in mice and AML patients. Significant differential expression was defined by Student’s *t* test *p* < 0.05; gene set enrichment analysis was conducted by GSEA, with a threshold on GSEA *p* value at 0.0005. **c**–**f** GSEA enrichment plots of selected oncogenic gene signatures. For each microarray dataset, all genes were sorted by the significance of differential expression between samples carrying mutant and wild-type *ASXL1* (denoted by *left* and *right arrows*). Gene sets were tested for overrepresentation at either side of the ranked list, of which the overrepresentation was measured by a running enrichment score (ES; *green curves*). Positive and negative ES represent enrichments in *ASXL1*-mutated and wild-type samples, respectively. Significance of an ES was assessed by random permutation of the gene list. **c**, **d** All of the five gene signatures showed *MN1*-transduction specific enrichments in mice. **e**, **f** Concordant enrichments were seen in AML patients, with no dependency on the expression of *MN1*. These oncogenic functions may partially account for the change in the threshold of *MN1*-driven engraftment in the presence of *Asxl1* mutation

Our mouse model provided an ideal platform to investigate the impacts of *Asxl1* mutation per se on global gene expression patterns, since the genetic backgrounds of our mice were far less complicated than those in human leukemia cells. In addition, we were interested in the mechanisms underlying the supportive role of *Asxl1* mutation in the engraftment of *MN1* overexpressing bone marrow cells. To these ends, we collected Lin^-^ marrow cells from *Asxl1* ^*tm/+*^ and wild-type control mice with or without *MN1* transduction (wild-type, *Asxl1* mutation, wild-type + *MN1* overexpression, and *Asxl1* mutation + *MN1* overexpression) for microarray analyses to explore differential gene expression patterns as well as molecular functions conferred by *Asxl1* mutation per se and/or its interplay with *MN1* overexpression. Of note, only 4.6% genes were significantly differentially expressed between *Asxl1*-mutated and wild-type cells (Fig. 7a; left bar); the number of perturbed functional gene sets was also very modest (3.2%, comparing *Asxl1* mutation vs. wild-type cells, Fig. 7b; left bar). However, the differences became obvious when comparing *Asxl1*-mutated cells overexpressing *MN1* vs. wild-type cells overexpressing *MN1* cells: up to 12.2% differentially expressed genes among all genes (Fig. 7a, second bar from the left), higher than that achieved by randomly shuffling the microarray dataset for 100 times (empirical *p* < 0.01), with correspondingly large scale of perturbed gene sets, up to 23.9% (Fig. 7b, second left bar). These data were consistent with our findings that *Asxl1* alone did not render obvious blood diseases in the mice but it might play a cooperative role with *MN1*.

To compare our mouse model with human disease, we profiled gene expression of leukemia cells from a total of 343 AML patients and compared the expression patterns between samples with (*N* = 50) and without (*N* = 293) *ASXL1* mutation. For 172 AML patients with higher *MN1* expression (above the median level), we also compared the expression patterns between those with (*N* = 29) and without (*N* = 143) *ASXL1* mutation. The disturbance of global gene expression profiles and gene sets related to *Asxl1* mutation were quite comparable and obvious in total cohort (Fig. 7a, right two bars) and in the subgroup of patients with higher *MN1* expression (Fig. 7b, right two bars).

### Gene set enrichment analysis revealed oncogenic functions perturbed by the interaction between *Asxl1* mutation and *MN1* overexpression

A deeper look into the lists of significantly differential gene sets derived by GSEA revealed a handful of crucial oncogenic functions perturbed by the interaction between *Asxl1* mutation and *MN1* overexpression. Hypoxia-related genes were implied to be relevant factors of leukemogenesis [33, 34]. In our data, while the expression of genes of a hypoxia signature did not show an overall change in mice with *Asxl1* mutation vs. wild-type littermates (GSEA *p* = 0.272; Fig. 7c), they were significantly co-upregulated in *Asxl1* ^*tm/+*^, compared to wild-type mice, in the presence of *MN1*-overexpression (*p* = 0.007; Fig. 7d). Similar enrichments were seen in signatures representing multipotent progenitors and hematopoietic stem cells, as well as genes related to oncogenic *KRAS* and *MEK*, in an *MN1*-dependent manner (all *p* values <0.0005 in *Asxl1* ^*tm/+*^ vs*.* wild-type mice transduced with *MN1*, Fig. 7d; compared with *p* > 0.05 in *Asxl1* mutation vs. wild-type mice without *MN1* overexpression, except for genes related to *MEK*, Fig. 7c, p value 0.012). Such positive enrichment toward *Asxl1* mutation was corroborated in AML patients, regardless of the abundance of *MN1* (all *p* values <0.0005; Fig. 7e and f). In aggregate, the logistic relationship between *Asxl1* mutation and *MN1* overexpression is summarized as (1) *Asxl1* mutation promoted engraftment of bone marrow cells in *MN1* overexpression background (Fig. 6e and Table 1); (2) AML patients with both *ASXL1* mutation and high *MN1* expression had inferior survival when compared with *ASXL1*-wild-type and high *MN1* expression (Fig. 6b); (3) In the background of *MN1* overexpression, *Asxl1* mutation in mice and in human AML patients was associated with upregulation of signatures of hematopoietic stem/progenitor cells and related to hypoxia, KRAS, and MEK pathways.

### ChIP-Seq analysis revealed *Asxl1* mutation-modulated binding profiles of H3K27me3

**Fig. 8:**
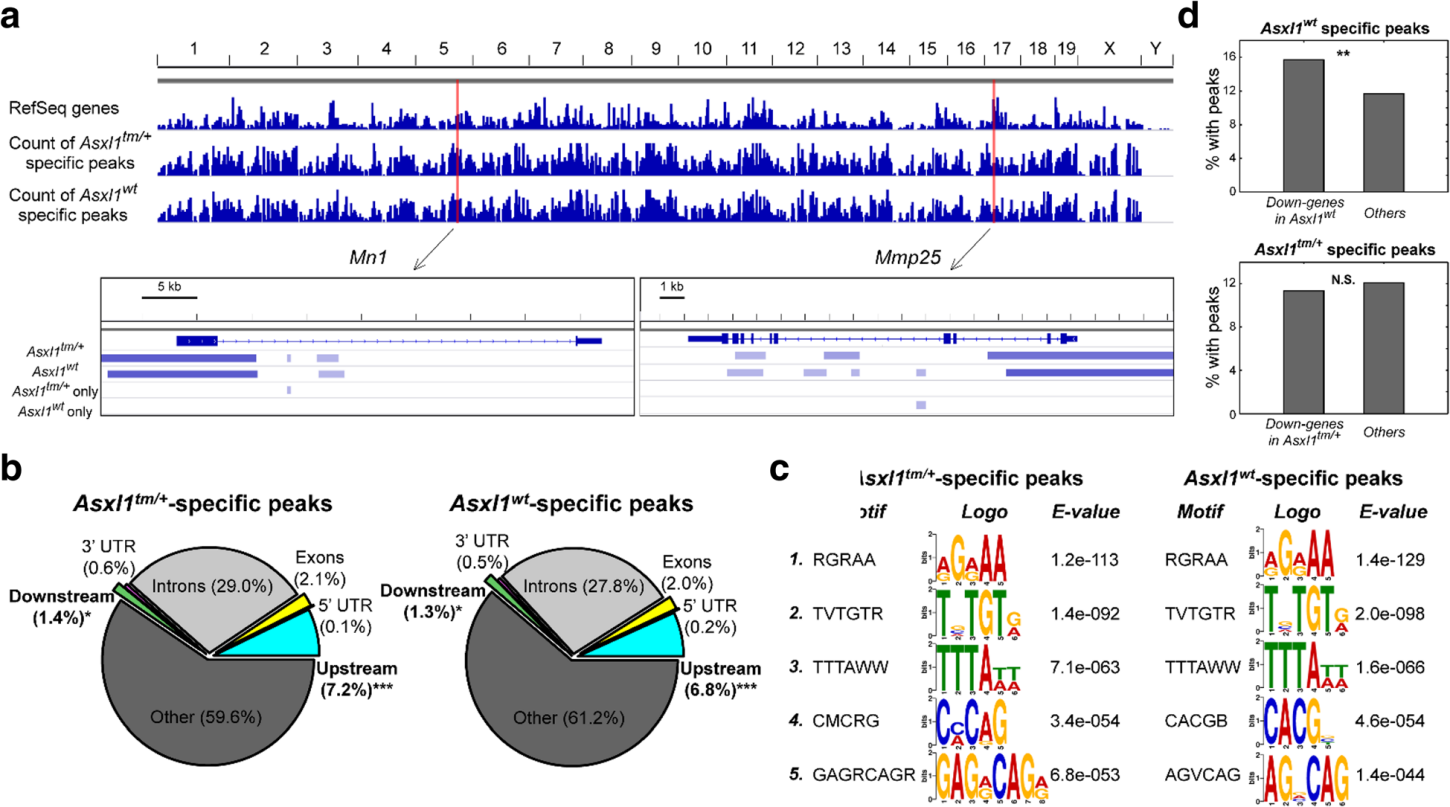
ChIP-Seq analysis of H3K27me3 in *Asxl1* ^*tm/+*^ and wild-type bone marrow cells. **a** Integrative Genome Viewer plots of *Asxl1* ^*tm/+*^ and wild-type specific peaks in the mouse genome. Bottom panels, enlarged plots of two examples. **b** Distributions of the *Asxl1* ^*tm/+*^ modulated peaks in gene regions. Upstream and downstream lengths were set at 5 k and 1 k bps., respectively. The specific H3K27 trimethylation peaks are significantly enriched in upstream and downstream regions. *, binomial test *p* < 0.05; ***, *p* < 0.001. **c** Top enriched motifs on the *Asxl1* ^*tm/+*^ modulated peaks reported by the MEME-ChIP web tool. **d** Proportions of significant downregulated genes in *Asxl1* wild-type (*upper panel*) and *Asxl1* ^*tm/+*^ (*lower*) cells harboring condition-specific H3K27me3 peaks. 15.69% of the *Asxl1* wild-type-specific downregulated genes harbored wild-type-specific H3K27 trimethylation peaks, while only 11.69% of other genes in *Asxl1* wild-type cells harbored wild-type-specific H3K27 trimethylation peaks. **, Fisher’s exact test *p* < 0.01; N.S., non-significant *p*

Several studies have linked functions of *Asxl1* mutation to H3K27me3, an inactive mark associated with transcriptional repression [19]. In order to investigate their interactions in our mouse model, we performed histone extraction followed by western hybridization to evaluate the global H3K27me3 levels in bone marrow cells. There was no significant difference in global H3K27me3 levels between *Asxl1* ^*tm/+*^ bone marrow cells and wild-type bone marrow cells (Additional file 1: Figure S6). Then, we analyzed if there was different global H3K27me3 pattern between *Asxl1* ^*tm/+*^ and wild-type Lin^-^ bone marrow cells via ChIP-Seq analysis. Comparing sequencing reads of ChIP products and input controls, we identified ~70 k H3K27me3-binding peaks in each of the *Asxl1* ^*tm/+*^ and wild-type samples. Of note, considerable proportions of them were *Asxl1* ^*tm/+*^ cells-specific (25,695; 37.0%) or wild-type (26,850; 37.7%) cells-specific. These peaks are highly concordant with gene loci in the mouse genome (Fig. 8a). *Mn1* harbored three H3K27me3-binding sites, of which one was *Asxl1* ^*tm/+*^-specific (Fig. 8a, left lower panel). We then analyzed the distribution of the condition-specific peaks in gene regions. Significant enrichment of peaks was found in upstream (within 5 k bps; 7.2 and 6.8% of *Asxl1* ^*tm/+*^- and wild-type-specific peaks, respectively; both *p* < 0.001) and downstream regions of gene bodies (within 1 k bps; 1.4%, *p* = 0.031; and 1.3%, *p* = 0.023, respectively) compared to randomly distributed peaks across the genome (Fig. 8b). Other genomic categories, such as 5’ and 3’ untranslated regions (UTRs) and exons, were not enriched (all *p* values >0.05), suggesting the preference of *Asxl1* mutation-associated H3K27me3 occupancy in gene regulatory regions.

To further investigate *Asxl1* mutation-modulated targets of H3K27me3, we analyzed enriched motifs on the peaks by the MEME-ChIP web tool, which performs motif discovery, enrichment, and visualization from DNA sequences of interest. Interestingly, while *Asxl1* ^*tm/+*^ and wild-type specific peaks do not overlap with each other, they carried very similar motifs: RGRAA, TVTGTR, and TTTAWW (all *E* values <0.001; Fig. 8c), indicating that the modulation of *Asxl1* mutation in H3K27me3 occupancy is independent of the binding motifs.

In order to investigate the effects of such selective targeting on gene expression and biological functions, we linked the peaks with gene expression microarrays. Lists of H3K27me peaks-associated genes with concordant significant downregulation are provided in Additional file 1: Table S1. Notably, the down-regulated genes in *Asxl1* wild-type cells were significantly associated with concordant *Asxl1* wild-type- specific H3K27m3 peaks (15.69% of the *Asxl1* wild-type-specific downregulated genes harbored concordant *Asxl1* wild-type-specific H3K27m3 peaks, compared to 11.69% of non-downregulated genes; Fisher’s exact test *p* = 0.001; Fig. 8d, upper panel). However, *Asxl1* ^*tm/+*^ cells-specific peaks were not significantly associated with down-regulated genes in *Asxl1* ^*tm/+*^ cells (Fisher’s exact test *p* = 0.52; Fig. 8d, lower panel), implying that in *Asxl1* mutated cells, the association between H3K27m3 and gene downregulation is disrupted when compared with *Asxl1* wild-type condition. Taken together, our ChIP-Seq data demonstrated distinct *Asxl1* mutation-modulated binding profiles of H3K27me3.

## Discussion

For the first time, we have demonstrated the pathophysiological functions of a “physiological” dose of *Asxl1* mutations in vivo and in vitro. In contrast to the previous studies with enforced overexpression of mutant ASXL1 protein in a background of two wild-type alleles of endogenous *Asxl1* [18, 19], our model facilitated investigation of a more clinically relevant *Asxl1* mutation.

In our study, we noted while *Asxl1* mutation promoted engraftment of *MN1*-overexpressing cells and showed increased colony formation and cobblestone area formation, the LSK cells bearing *Asxl1* mutation had inferior repopulation capacities when compared with wild-type cells in vivo. This counterintuitive observation could be explained by two possibilities: (1) in our in vivo transplantation assays (Figs. 3 and 4), we assessed the activities of HSCs. But *MN1* overexpression targeted committed progenitor cells, not HSCs [35]. This may explain the discrepancies between these experimental results; (2) in serial transplantation, the marrow stem cells were taken and expanded in a previously irradiated microenvironment, not normal hematopoietic niche. Spangrude et al. have shown a vastly inferior repopulation capacity of LSK cells repeatedly exposed to such perturbed microenvironment [36]. Such radiation perturbation on microenvironment was absent in colony formation and cobblestone area formation assays, and less severe in *MN1* overexpressing cell transplantation assays. We could not rule out the possibility that *Asxl1* mutant cells were particularly susceptible to this factor, thus showing decreased repopulation capacity in serial transplantation assays, while similar phenomenon was not shown in the other assays without serial irradiation. Moreover, Kamminga et al. showed that although a gradual decrease of the percentage of LSK cells was observed when LSK cells were used as donor cells in serial transplantation, only minor decrease was observed for the clonogenic CAFC activity of the purified cells [37]. These results suggested that in vivo repopulation ability of LSK cells might be affected by residue host cells or competitor cells. They also highlighted the limitation of current animal assays to detect the “real” in vivo hematopoietic stem cell activities.

In our model, *Asxl1* G643WfsX12 mutation did not lead to leukemia or other blood malignancies in a 18-24-month observation period, indicating that a physiological dose of *Asxl1* G643WfsX12 was not sufficient for leukemogenesis. Nevertheless, the mutation could enhance engraftment of *MN1* overexpressing cells, suggesting that *Asxl1* mutation could function as a cooperative hit of *MN1* overexpression to promote the engraftment of bone marrow cells. This is consistent with the clinical observation that *ASXL1* mutant burden often increases in disease progression and mutations in *ASXL1* [38–41], as well as other genes encoding epigenetic modifiers, were often acquired early in the disease and were almost never found in isolation [42].

Gene expression microarray and GSEA showed limited difference between *Asxl1* ^*tm/+*^ and wild-type control bone marrow cells under steady state, consistent with our observation that *Asxl1* ^*tm/+*^ mice did not develop obvious blood diseases. In *MN1* overexpression background, the expression patterns and physiological pathways between *Asxl1* mutation and wild-type became distinctive (Fig. 7), implying the promoting effects of *Asxl1* mutation on *MN1* overexpression-induced engraftment of bone marrow cells. The high number of differentially expressed genes and perturbed biological pathways in human *ASXL1*-mutated versus wild-type AML cells demonstrated a far more complicated milieu in human AML cells compared with mice HSPCs with *Asxl1* mutation per se (Fig. 7a, b).

From our microarray studies, we found that *Asxl1* mutation alone in mice had little effects on both gene expression profiles and biological pathways, while the perturbation became obvious in the presence of *MN1* overexpression. How *MN1* overexpression augments the genomic effects of *Asxl1* mutation is not completely defined in our study, but we found that *Asxl1* mutation plus *MN1* overexpression, but not *Asxl1* mutation alone, was associated with enrichment of signatures representing multipotent progenitors and hematopoietic stem cells, as well as genes related to oncogenic *MEK*.

Hypoxia-related genes are considered critical for the survival of leukemia initiation cells [34, 43]. The enrichment in hematopoietic stem cell and multipotent progenitor gene sets further implies the supporting function of *ASXL1* mutation in blood malignancies. *KRAS* is considered relevant in leukemia formation [44–46]; the enrichment in *KRAS* gene set confers the possibility that mutant ASXL1 act as a cofactor in disease development. MAPK/ERK pathway is crucial for hematopoiesis and aberrant MAPK/ERK pathway is associated with cancer formation [47]. RAS signaling are also considered to be involved in AML transformation at both genetic and epigenetic levels [48]. Bone marrow cells of our mouse model were supposed to have *Asxl1* mutation alone, but in *ASXL1*-mutated human AML cells, we expected there were additional genetic perturbations. One of the advantages of our mouse model was that it enabled us to interrogate the functions of *Asxl1* mutation per se, in a “simpler” genetic background. This was probably why we saw different biological effects of *MN1* overexpression between BM cells in our mouse model and human AML cells with more complex genetic background.

Since *Asxl1* has been considered to be associated with the regulation of H3K27me3, we performed ChIP-Seq to investigate the alteration of global H3K27me3 pattern in *Asxl1* ^*tm/+*^ bone marrow cells. Considerable numbers of H3K27me3 peaks specific to *Asxl1* ^*tm/+*^ and to wild-type bone marrow cells were noted and preponderantly located within 5 k upstream and 1 k downstream of gene bodies. These results indicate that *Asxl1* mutation can modulate the global pattern of histone methylation in a non-random manner, preferentially immediate to the gene bodies. In addition, in *Asxl1* mutated cells, the correlation between H3K27m3 and gene down-regulation appears attenuated when compared with *Asxl1* wild-type context, suggesting functional implications of Asxl1 functions in H3K27me3 modulation. Taken together, our systematic analyses unveiled crucial oncogenic functions perturbed by the interplay between *Asxl1* mutation and *MN1* overexpression that may partially account for the cooperative role of *Asxl1* mutations in *MN1*-associated leukemia in human and mouse settings and the functional impacts of *ASXL1* mutation in human AML.

## Conclusions

Taken together, for the first time, our study reveals the in vitro and in vivo effects of a “physiological” dose of *Asxl1* mutation. Although mutant *Asxl1* does not act as a sufficient driver in blood malignancies, it facilitates engraftment of cells overexpressing *MN1*. Our study also enlightens the effects on global H3K27m3 profiles by *Asxl1* mutation and several potential biological pathways underlying mutant *ASXL1*.

## Additional file

Additional file 1: Supplementary methods, figures and table. (PDF 913 kb)

## Abbreviations

AML: Acute myeloid leukemia
*Asxl1*^*tm/+*^: *Asxl1* G643WfsX12 heterozygous mice
BMC: Bone marrow cells
CAFC: Cobblestone-area-forming cell assay
CFC: Colony-forming cell
ChIP-Seq: Chromatin immunoprecipitation sequencing
CMP: Common myeloid progenitors
CRU: Competitive repopulating unit assay
GMP: Granulocyte-monocytic progenitors
GSEA: Gene Set Enrichment Analysis
H3K27me3: Tri-methylation of Histone 3 at lysine 27
HPC: Hematopoietic progenitors
HSC: Hematopoietic stem cells
HSPC: Hematopoietic stem cells and progenitors
LK: lin^-^c-Kit^+^Sca-1^-^ cells
LSK: lin^-^c-Kit^+^Sca-1^+^ cells
LSK^-^: lin^-^c-Kit^-^Sca-1^+^ cells
LTC-IC: Long-term culture-initiating cells
MDS: Myelodysplastic syndrome
MEP: Megakaryocyte-erythroid progenitors
WT: Wild-type control mice
