# Supplementary material for: The distinct biological implications of Asxl1 mutation and its roles in leukemogenesis revealed by a knock-in mouse model
Source: J Hematol Oncol. 2017 Jul 11;10:139. doi: 10.1186/s13045-017-0508-x (PMC5504705; doi:10.1186/s13045-017-0508-x)
Supplement: Additional file 1: — Supplementary methods, figures and table. (PDF 913 kb) [file 13045_2017_508_MOESM1_ESM.pdf]

Supplementary Material and Methods

Mouse cobblestone-area-forming cell assay

OP9 cells, a mouse marrow stromal cell line, were harvested 1 day prior to experiments using standard trypsinization protocol and treated with 10 mg/mL mitomycin C for 2 hours to inhibit proliferation. Then OP9 cells were seeded in 6-well plates and reached 100% confluent. On the day of the experiment, equal number of *Asxl1*$^{tm/+}$ and wild-type bone marrow cells were seeded on OP-9 stromal layer cells. After 1 week, colonies growing underneath the stromal layer were counted on an inverted microscope.

Colony-forming cell assay

In the initial plating, bulk bone marrow cells were plated in MethoCult™ GF M3434 (Stemcell technologies, BC, Canada) on 35mm culture dish according to user manual and the seeding concentration was 5000 cells per dish. After culture for 1 week, each type of colony was identified and counted, then MethoCult™ were dissolved by 4 times volume of complete IMDM medium and cells were harvested by regular centrifugation. Seeding concentration of second plating was 5000 cells per dish and colonies were cultured for 1 week before identification and colony counting. Colonies were harvested as previously described and seeding concentration for third plating was 3000 cells per dish. Colonies of third plating were identified and counted after another 1-week culture period.

Long term culture-initiating cell (LTC-IC) assay

Mouse bone marrow cells were seeded on the OP9 stromal layer in 3-fold dilution from 1215 to 5 cells per well in a 96-well plate, each with 10-20 replicates. Bone marrow cells and stromal layers were cultured in M5300 medium with 10^{-6} M hydrocortisone (both from Stemcell Technologies, BC, Canada) and half of the medium was replaced with fresh medium every week for 5 weeks. Subsequently, all medium was replaced with MethoCult™ GF M3434 (Stemcell technologies, BC, Canada). Number of wells which had colonies were scored and long term culture-initiating cell frequency were calculated by L-Calc software (Stemcell technologies, BC, Canada).

Mouse bone marrow transplantation and competitive repopulating unit assay (CRU)

Nucleated mouse bone marrow cells isolated from wild-type and *Asxl1* knock-in mice were used for bone marrow transplantation. B6.SJL-Ptprc^{a}pepc^{b}/BoyJ (CD45.1) recipient mice were lethally irradiated and then donor cells (CD45.2) were administered by retro-orbital injection within 24 hours. Peripheral blood was collected and the population size of CD45.1 and CD45.2 peripheral blood cells were analyzed to evaluate the reconstitution of the hematopoietic system every 4 weeks.

In competitive repopulating unit assay, a fixed number of CD45.1 whole bone marrow cell were used as helper cells and CD45.2 *Asxl1* knock-in or wild-type $Lin^{-}Sca\text{-}1^{+}c\text{-}Kit^{+}$ (LSK) bone marrow cells were used as the test cells. Test cells were mixed with CD45.1 helper cells and transplanted into lethally irradiated CD45.1 recipient. Peripheral blood was collected and the population size of CD45.1 and CD45.2 cells in peripheral blood were analyzed every 4 weeks. Recipients were sacrificed 16 weeks after transplantation and the frequency of $CD45.2^{+}$ HSPCs in bone marrow were evaluated by flow cytometry.

FACS analysis and Cell sorting

For FACS analysis, single cell suspension from either cultured cells or mouse hematopoietic organs (bone marrow or spleen) were stained with FITC, APC or PE-labeled anti-mouse monoclonal antibodies. The population size of long-term hematopoietic stem cells ($Lin^{-}Sca\text{-}1^{+}c\text{-}Kit^{+}CD150^{+}CD48^{-}$), short-term hematopoietic stem cells ($Lin^{-}Sca\text{-}1^{+}c\text{-}Kit^{+}CD150^{+}CD48^{+}$), multipotent progenitors ($Lin^{-}Sca\text{-}1^{+}c\text{-}Kit^{+}CD150^{-}CD48^{+}$), common myeloid progenitors ($Lin^{-}Sca\text{-}1^{-}c\text{-}Kit^{+}CD34^{+}Fc\gamma R^{lo}$), granulocyte-monocytic progenitors ($Lin^{-}Sca\text{-}1^{-}c\text{-}Kit^{+}CD34^{+}Fc\gamma R^{hi}$), and megakaryocyte-erythroid progenitors ($Lin^{-}Sca\text{-}1^{-}c\text{-}Kit^{+}CD34^{-}Fc\gamma R^{lo}$) were acquired on BD LSRFortessa, BD FACSAriaIII or BD FACSVerse. For cell sorting, desired mouse bone marrow cells were sorted using a BD FACSAriaIII multicolor cell sorter. Data were analyzed using FlowJo™ software (FlowJo, Ashland, OR).

Retroviral transduction of *MN1*

Retrovirus carrying *MN1* gene was generated by transient transfection of Plat-E retroviral packaging cell line (Cell Biolabs, San Diego, CA). Three days after transfection, the retrovirus was harvested by collecting conditioned medium of Plat-E cells. The conditioned medium was filtered through 0.22 µm syringe filter and concentrated via Amicon Ultra-15 Centrifugal Filter Units, then the virus concentrate was applied to the mouse bone marrow cultured in StemSpan SFEM (Stemcell technologies, BC, Canada) complete medium with 5 µg/mL protamine sulfate, 20 ng/mL recombinant mouse SCF, 10 ng/mL recombinant mouse IL-3 and 20 ng/mL recombinant mouse IL-6. After 72 hours, Lin^{-} vital cells with green fluorescence (GFP^{+}) were sorted for further experiments.

Microarray analysis

Mouse bone marrow cells were harvested from mice between 8-12 weeks old. Lin^{-} bone marrow cells were sorted for RNA extraction. Gene expression profiling was performed using Affymetrix Mouse Transcriptome Array 1.0 (Affymetrix, Santa Clara, CA) or Mouse Ref-8 BeadChip (Illumina, San Diego, CA). All microarray data were quantile normalized to eliminate inter-sample biases. Paired and unpaired two-

sample Student's *t*-tests were used to assess the differences between samples with and without *Asxl1* mutations; $p<0.05$ was set as threshold for significance.

In order to investigate the functional relevance of *ASXL1* mutation, we used the Gene Set Enrichment Analysis (GSEA) software to analyze the enrichment of KEGG pathways, Gene Ontologies, oncogenic signatures, and immunologic signatures in the global gene expression profiles. Briefly, all genes were ranked according to their significance assessed by *t*-tests, with two ends representing the most significantly up-regulated genes in samples with mutant and wild-type *ASXL1*, respectively, and tested for overrepresentation of member genes of a gene set at either side of the list. Significance of overrepresentation was assessed against random permutations with respect to genes.

Histone extraction and western hybridization

Histone of either *Asxl*$^{tm/+}$ and wild-type mouse bulk bone marrow cells were extracted by histone extraction kit according to user manual (Abcam, UK) and then standard western hybridization was performed subsequently for immunostaining. Anti-H3K27me3 (Genetex, USA) was used to determine H3K27me3 amount and anti-Histone H3 (Upstate, Germany) was used to detect total histone H3 as internal control.

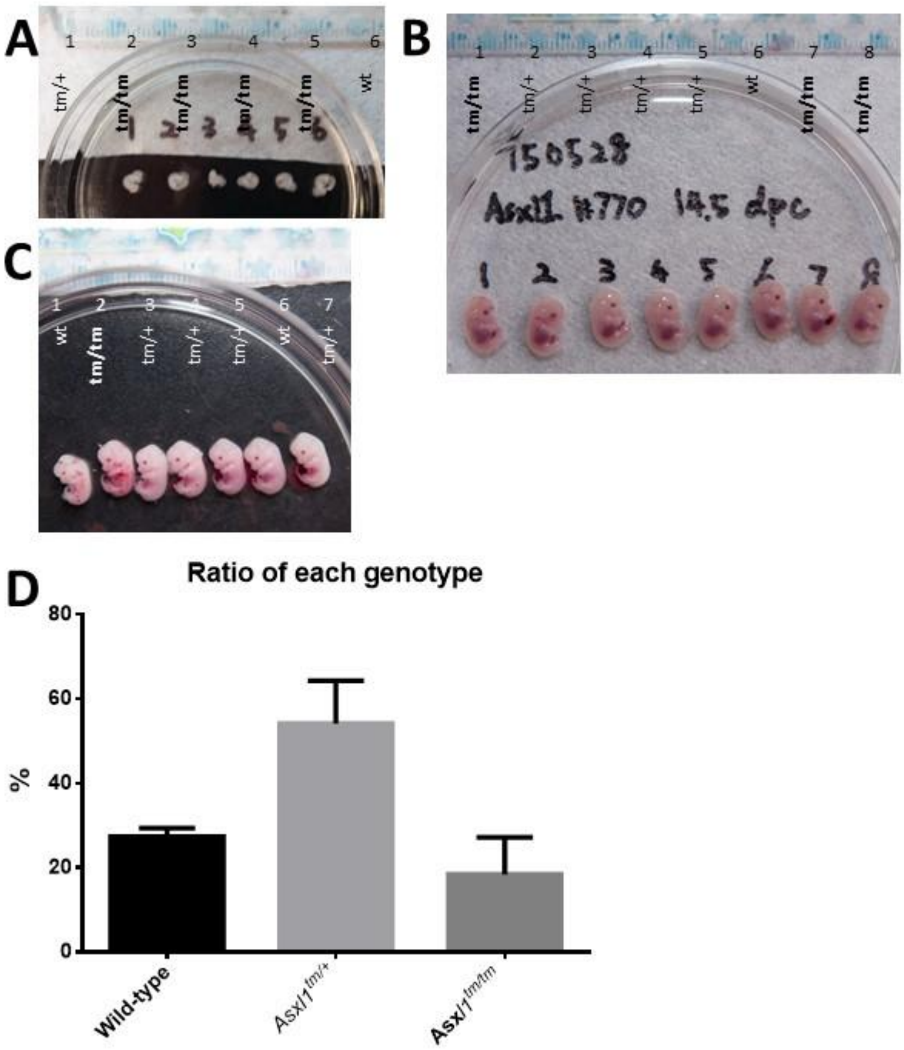

Supplementary figure S1. Genotype of newborn mice under heterozygous mate heterozygous breading plane. (A) Genotype of each pup at 10.5 dpc. (B) & (C) Genotype of each pup at 14.5 dpc. (D) Ratio of each genotype after delivery (N=22 in 3 litters).

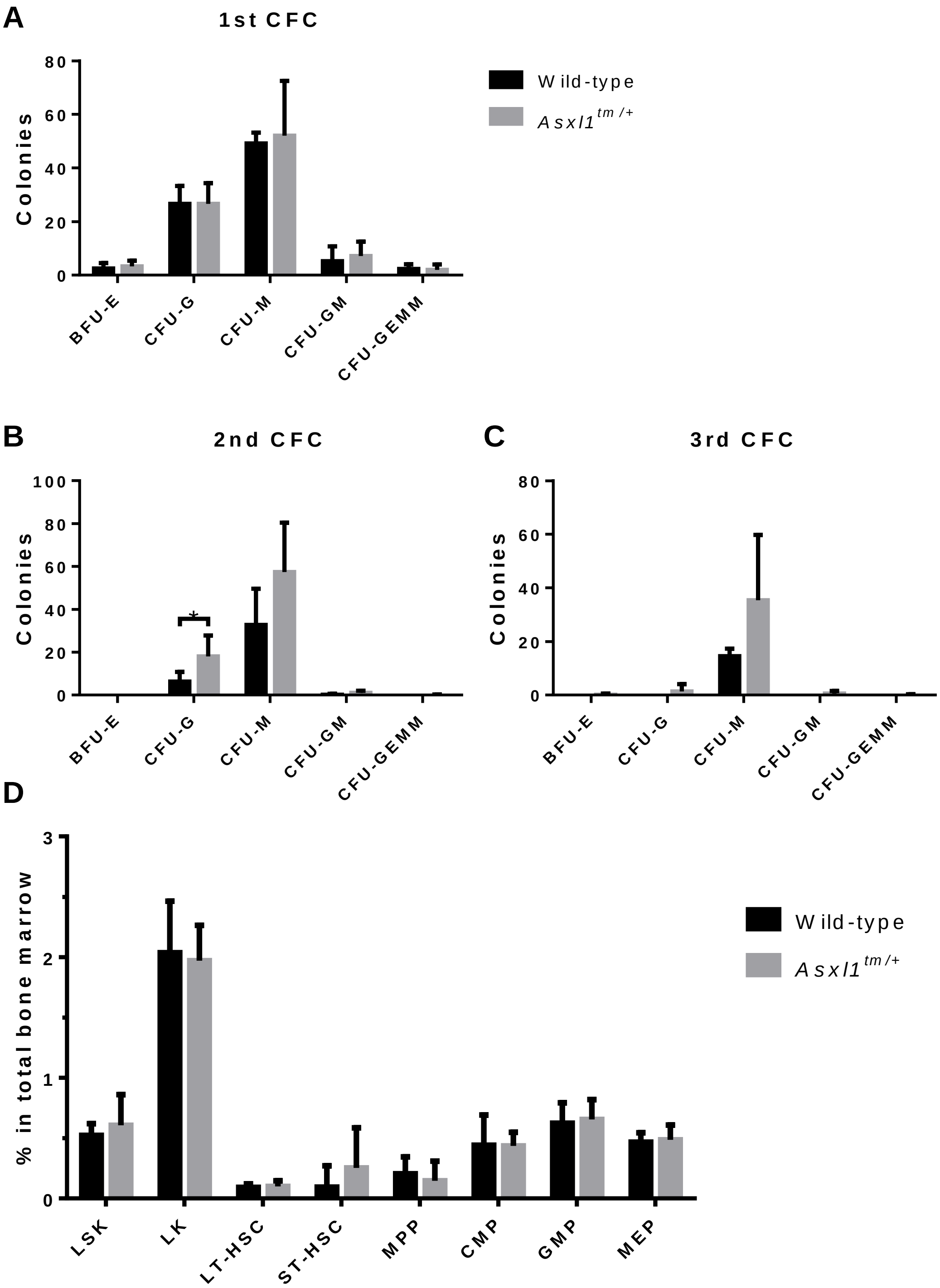

Supplementary figure S2. Detail results of colony assay and population frequency of hematopoietic stem cells and progenitors in bone marrow. (A) Number of each colony type at the first CFC assay. (B) Number of each colony type at the second CFC assay. (C) Number of each colony type at the third CFC assay. (D) Frequency of hematopoietic stem cells and progenitors in *Asxl1*$^{tm/+}$ and wild-type mice bone

marrow.

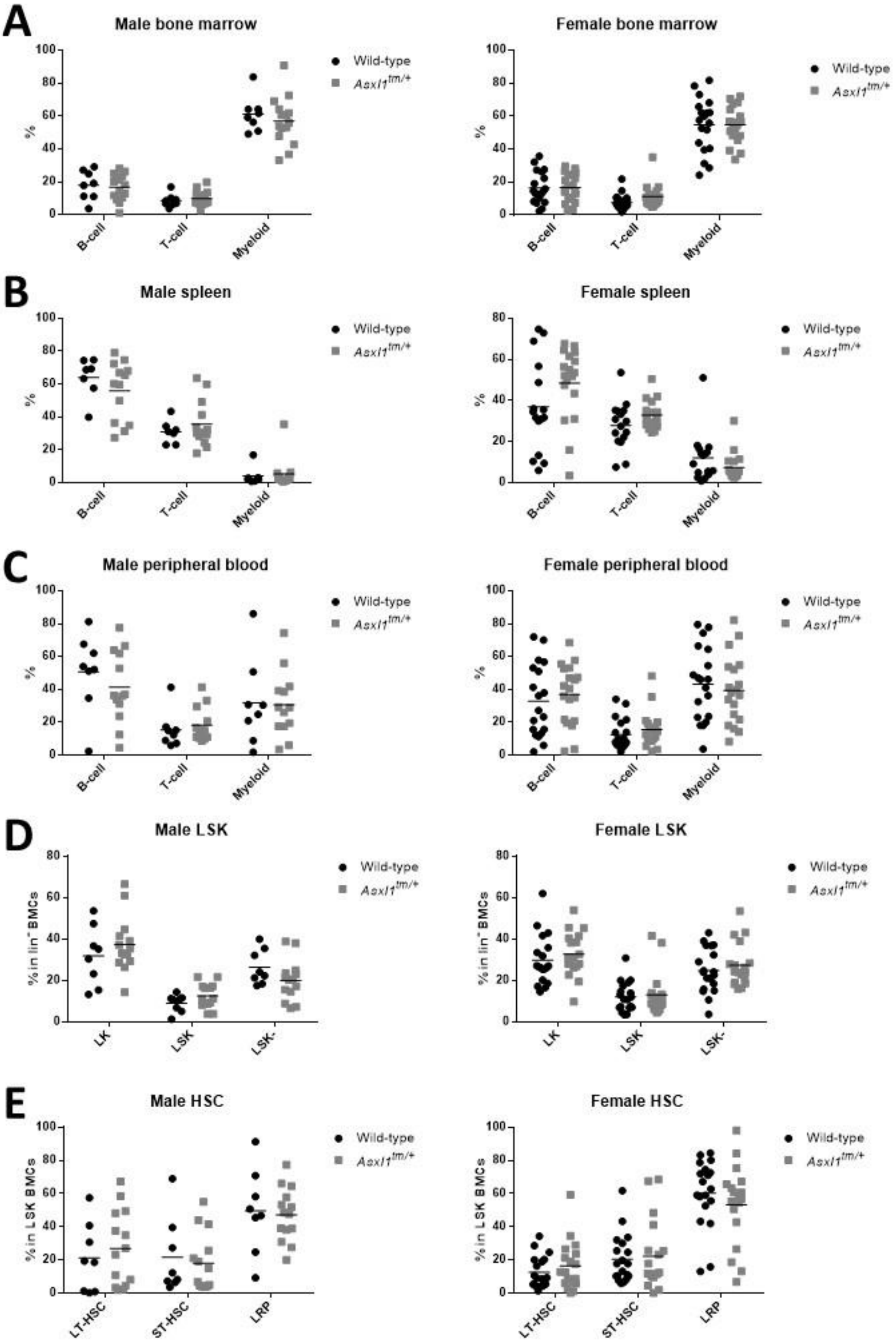

Supplementary figure S3. FACS analysis of old *Asxl1*$^{tm/+}$ and wild-type mice's bone marrow cells (BMCs), spleen cells and peripheral blood cells. (A) Proportion of B-cell, T-cell and myeloid in old mice's bone marrow (Right panel: female mice; left panel: male mice). (B) Proportion of B-cell, T-cell and myeloid in old mice's spleen

(Right panel: female mice; left panel: male mice). (C) Proportion of B-cell, T-cell and myeloid in old mice's peripheral blood (Right panel: female mice; left panel: male mice). (D) Composition of HSPC in old mice's bone marrow (Right panel: female mice; left panel: male mice). (E) Composition of HSC in old mice's bone marrow (Right panel: female mice; left panel: male mice).

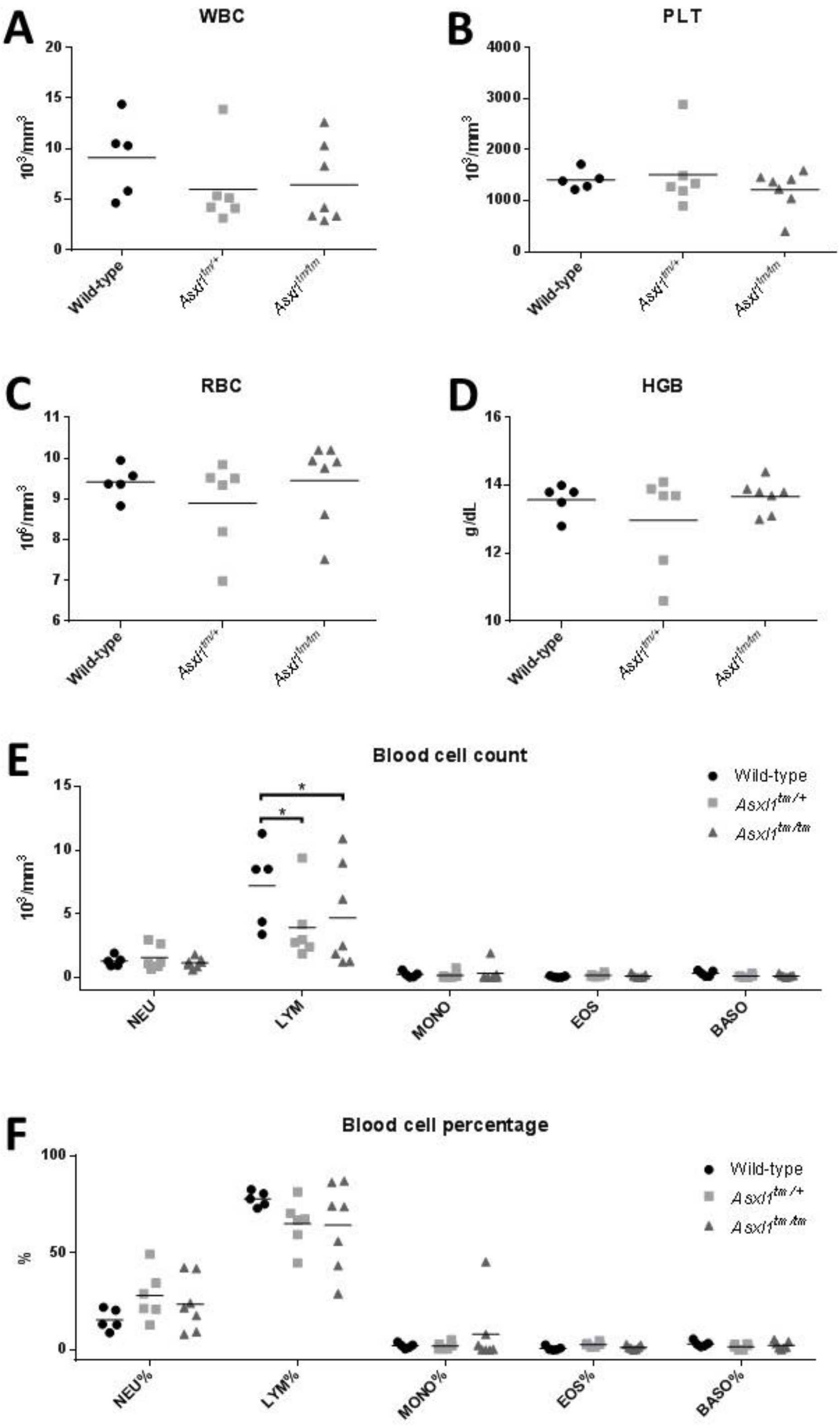
A
WBC
10^3/mm^3
Wild-type
Asxl1tm/+
Asxl1tm/tm
B
PLT
10^3/mm^3
C
RBC
10^6/mm^3
D
HGB
g/dL
E
Blood cell count
10^3/mm^3
NEU
LYM
MONO
EOS
BASO
Wild-type
Asxl1tm/+
Asxl1tm/tm
F
Blood cell percentage
%
NEU%
LYM%
MONO%
EOS%
BASO%

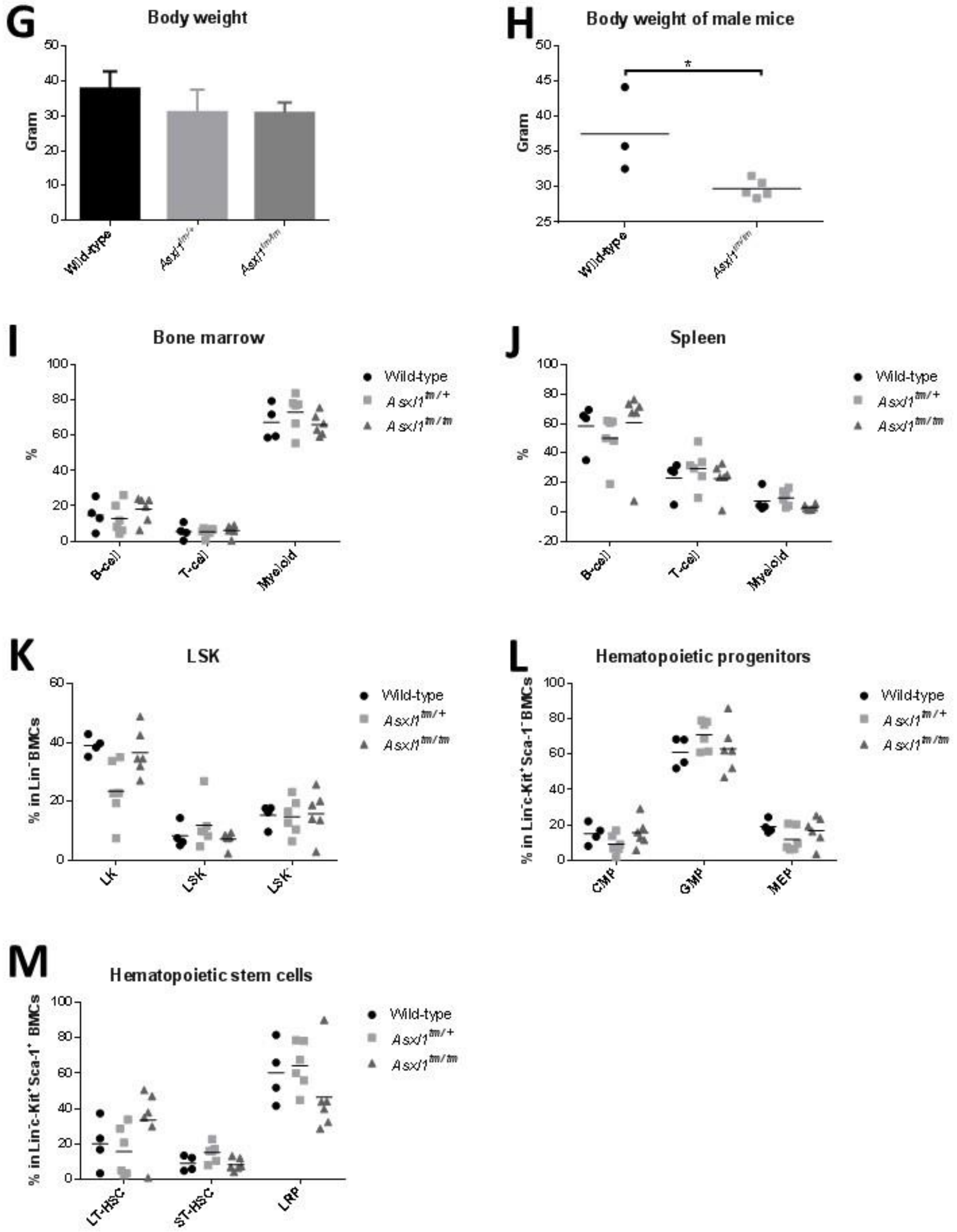

Supplementary figure S4. Hemogram of old *Asxl1*$^{tm/tm}$ mice cohort. (A) White blood cell counts of 3 genotypes (Wild-type N=5, *Asxl1*$^{tm/+}$ N=6, *Asxl1*$^{tm/tm}$ N=7). (B) Platelet counts of 3 genotypes (Wild-type N=5, *Asxl1*$^{tm/+}$ N=6, *Asxl1*$^{tm/tm}$ N=7). (C) Red blood cell counts of 3 genotypes (Wild-type N=5, *Asxl1*$^{tm/+}$ N=6, *Asxl1*$^{tm/tm}$ N=7). (D) Hemoglobin value of 3 genotypes (Wild-type N=5, *Asxl1*$^{tm/+}$ N=6, *Asxl1*$^{tm/tm}$ N=7). (E) and (F) Neutrophil, lymphoid, monocyte, eosinophil and basophil counts and

percentages of 3 genotypes (Wild-type N=5, *Asxl1*$^{tm/+}$ N=6, *Asxl1*$^{tm/tm}$ N=7). (G) Average body weight of every mouse in this cohort (Wild-type N=5, *Asxl1*$^{tm/+}$ N=6, *Asxl1*$^{tm/tm}$ N=7). (H) Male homozygous mice were smaller than male wild-type mice. Only male wild-type and homozygous had sufficient numbers to do statistics. (I) B-cell, T-cell and myeloid frequencies in bone marrow (Wild-type N=4, *Asxl1*$^{tm/+}$ N=6, *Asxl1*$^{tm/tm}$ N=6). (J) B-cell, T-cell and myeloid frequencies in spleen (Wild-type N=4, *Asxl1*$^{tm/+}$ N=6, *Asxl1*$^{tm/tm}$ N=6). (K) Frequencies of hematopoietic precursors in Lin^{-} bone marrow cells (Wild-type N=4, *Asxl1*$^{tm/+}$ N=6, *Asxl1*$^{tm/tm}$ N=6). (L) Frequencies of hematopoietic progenitors in Lin^{-}c-Kit^{+}Sca-1^{-} bone marrow cells (Wild-type N=4, *Asxl1*$^{tm/+}$ N=6, *Asxl1*$^{tm/tm}$ N=6). (M) Frequencies of hematopoietic stem cells in Lin^{-}c-Kit^{+}Sca-1^{+} bone marrow cells (Wild-type N=4, *Asxl1*$^{tm/+}$ N=6, *Asxl1*$^{tm/tm}$ N=6).

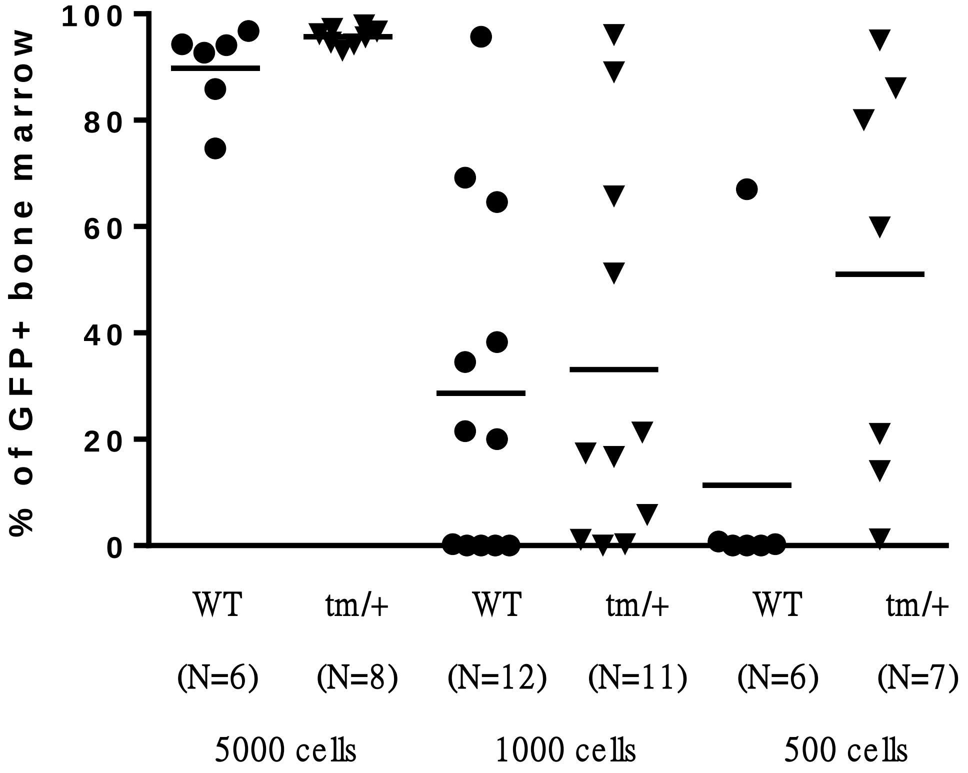

Supplementary figure S5. Serial dilution transplantation assays showed that while transduced by *MN1*, higher proportion of *Asxl1*$^{tm/+}$ bone marrow cells carried engraftment ability compared to the wild-type controls (WT=wild-type and tm/+=*Asxl1*$^{tm/+}$).

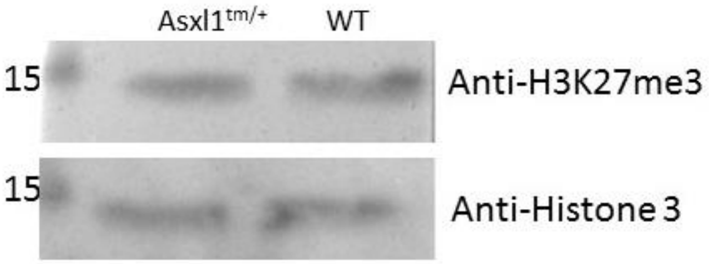

Supplementary figure S6. *Asxl1*$^{tm/+}$ did not significantly alter global H3K27me3 level in bulk bone marrow cells.

Supplementary Table S1. Lists of H3K27m3 peaks-associated genes with concordant downregulation.

Asxl1 wild-type specific H3K27me3 peaks with concordant downregulation in *Asxl1* wild-type cells (118 genes)				*Asxl1* mutant specific H3K27me3 peaks with concordant downregulation in *Asxl1* mutant cells (112 genes)			
1200007C13RIK	DAPL1	LRFN5	SAMD4	1700036G14RIK	GM13746	MDGA1	SERPINB1B
1700016D06RIK	DGKK	LY6F	SEC16B	1700066B19RIK	GM13807	MED12L	SERPINH1
3300002I08RIK	DMD	MAP3K15	SEMA4A	1700112J16RIK	GM14110	MKRN3	SLC41A2
4632411P08RIK	DUSP9	ME1	SETBP1	1700113H08RIK	GM14319	MOB1B	SMT3H2-PS4
4930407I10RIK	ECE1	MMP25	SH2D1B1	4933402D24RIK	GM15458	N-R5S12	STK36
4930432B10RIK	EFCAB6	MPPED1	SLC16A5	ABCA1	GM16581	NEB	SYT4
4930471D02RIK	EPHA10	MYH1	SLC29A4	ADAM5	GM22069	NR1H4	TBC1D21
4932411E22RIK	FABP4	MYO16	SLC45A1	AGTPBP1	GM23175	NRCAM	TBPL2
4933436E23RIK	FKBP9	NCAM1	SLC4A10	APOH	GM23816	OLFR1	TCL1B3
9530085P06RIK	FLG	NHSL1	SLC7A2	AW495222	GM24601	OSBPL6	TMED6
9830107B12RIK	FMN1	NPNT	SORCS2	AY512915	GM24981	PAMR1	TMEM130
A230107N01RIK	FSIP2	OLFR53	SPATS2L	CACNA2D2	GM6117	PBX1	TRIM66
A830019P07RIK	GAS7	OLFR553	SQRDL	CCDC27	GM7237	PBX3	TRIM71
ACAN	GM10650	OLFR685	SRRM4OS	CCR4	GM7420	PDE4DIP	U90926
AMPH	GM13307	PDE1A	STOX2	CHIA1	GPR174	PIK3C2G	UGGT2
ANPEP	GM15433	PELI2	SV2B	CHN1OS3	GSE1	PLEKHS1	ULK4
AOX1	GM16511	PGAP1	SYTL2	CSGALNACT1	HAPLN3	POSTN	VWA2
ATP1B4	GM22152	PLCB1	TAGLN	CXCL11	HGFAC	PPP3R1	VWA3B
AY512931	GM22284	PPIC	TGFBI	DHRS7C	HIST1H1B	PRSS12	ZBTB8B
BCKDHB	GM25216	PPM1A	TMEM132C	DNAH17	IL13RA1	PRSS43	ZC3H12B
BMX	GM26395	PRAP1	TMEM233	DNAH8	IL1A	PTOV1	ZFP112
C1QTNF1	GM3248	PRM1	TRIM2	DRP2	IQSEC3	RAB37	ZFP385B
CAPS2	GM3558	PTPRM	TRPC1	ELFN1	KCNIP1	RAB3GAP2	
CCDC60	GM7073	PXDC1	USP51	FAM160A2	KCNK6	RAI2	
CCDC85B	GM94	RDH18-PS	VSNL1	FAM81A	KCNU1	RHOX2D	
CCL27A	GPRIN2	RET	WDR93	FGF5	L3MBTL4	RNLS	
CDKL4	IL3	RGS20	ZDHHC2	FSCN1	LIN28B	RORA	
CTNND2	ILDR1	RHOD	ZFP641	GM11770	LINGO2	ROS1	
CYP2C70	INADL	RIAN		GM12153	LIPO1	RPL31-PS17	
D930032P07RIK	LGR4	RNF182		GM13417	MAP3K12	S100A3	
